# The Genuine Works of Hippocrates

**Published:** 1891-12

**Authors:** 


THE GENUINE WORKS OF HIPPOCRATES. Translated from the Greek, with a Preliminary Discourse and Annotations. By FRANCIS ADAMS, LL. D., Surgeon. Octavo, 766 pages, extra muslin, gilt top, price, $5.00. New York: William Wood & Company. 1891.

Every physician should commune with the fathers of medicine, and particularly with the great Hippocrates, who may be justly regarded as the most learned of them all. Very few physicians in active practice can command sufficient time to read medical classics in their original tongue. Hence, it is a pleasure and a profit to be able to read these works in a modern language. The council of the Sydenham Society did wisely in selecting Dr. Adams to translate such of the most important works of the great father of medicine as appear in the volume before us. The translator has illustrated his labor by annotations, arguments, and such-like methods which are given in the way of foot notes. This adds much to the interest of the book and serves to explain many obscure points, because we thus are able to obtain the opinion of a scholar upon doubtful and technical phrases. The publishers have presented this book as an *edition de luxe*, bound in mottled linen boards with gilt borders and untrimmed edges, which, together with the excellent paper and press-work, make it one of the most beautiful that have been issued for physicians by any American press.

